# STAT1 as a potential prognosis marker for poor outcomes of early stage colorectal cancer with microsatellite instability

**DOI:** 10.1371/journal.pone.0229252

**Published:** 2020-04-10

**Authors:** Atsushi Tanaka, Yihua Zhou, Makiko Ogawa, Jinru Shia, David S. Klimstra, Julia Y. Wang, Michael H. Roehrl

**Affiliations:** 1 Department of Pathology, Memorial Sloan Kettering Cancer Center, New York, NY, United States of America; 2 Department of Pathology, Graduate School of Medicine, University of Tokyo, Tokyo, Japan; 3 Human Oncology and Pathogenesis Program, Memorial Sloan Kettering Cancer Center, New York, NY, United States of America; 4 ICU Department, Second Affiliated Hospital of Nanchang University, Nanchang, Jiangxi, China; 5 Curandis, New York, NY, United States of America; Leiden University Medical Centre, NETHERLANDS

## Abstract

Proteomic analyses indicate that STAT1 protein (signal transducer and activator of transcription 1 or transcription factor ISGF-3 components p91/p84) is upregulated in some colorectal cancers. This study examined 736 colorectal cancer patients for the expression of STAT1 protein in tissue specimens, including 614 early stage patients and 122 advanced stage patients. Tissue microarrays were constructed, and STAT1 expression was examined by immunohistochemistry and scored semi-quantitatively. Among all cases, 9% of cases displayed high levels of cytoplasmic expression of STAT1 and 15% of cases had positive nuclear expression. Based on statistical analyses of a cohort of 559 early stage patients with survival data and no neoadjuvant therapy, we found that high levels of cytoplasmic expression of STAT1 correlated with shorter survival time in early stage colorectal cancer, particularly of the microsatellite instability (MSI) subtype. Additional analysis of a 244-case cohort of colorectal cancers from the Cancer Genome Atlas found that STAT1 gene expression correlated positively with PD-L1 (CD274) and PD-1 (PDCD1) but had no correlation with KRAS or BRAF mutation status. STAT1 expression showed no clear correlation with any of the 4 clinical diagnostic markers of mismatch repair, MLH1, MSH2, MSH6, and PMS2, suggesting its potential as an independent outcome marker for MSI cancers. Our findings suggest that STAT1 may be used as a potential prognostic protein marker for stratifying the outcome risk of early stage MSI colorectal cancer.

## Introduction

Colorectal cancer (CRC) is among the most prevalent malignant tumors and a leading cause of cancer deaths worldwide [1]. CRC can be successfully treated if discovered at an early stage, with 5-year overall survival rate approaching 90% [2, 3]. Risk assessment of early stage CRC is particularly critical because it determines whether adjuvant chemotherapy or targeted molecular therapy should be administered. However, early stage risk assessment is challenging because of a lack of reliable prognostic molecular biomarkers. Morphological and clinical features such as poorly differentiated histology, lymphovascular invasion, perineural invasion, bowel obstruction, localized perforation, and positive margins have been reported to worsen the prognosis of CRC [4–6], yet none of these has allow optimal stratification for adjuvant therapy in resected, early stage carcinomas. Molecular biomarkers with more precise prognostic value, preferably with an underlying functional pathophysiologic rationale, would enable us to better stratify risk of early stage CRC after resection and more accurately select patients for additional therapy, while avoiding overtreatment in low-risk patients.

CRC is heterogeneous and often sub-classified as subtypes with either microsatellite stability (MSS) or microsatellite instability (MSI). MSI, also commonly referred to as MSI-high, results from deficient mismatch repair and serves as a screening tool for Lynch syndrome [7]. Microsatellites are regions of repeated DNA sequences distributed throughout the genome. When mismatch repair does not work properly, microsatellites are prone to replication errors and may become longer or shorter, resulting in instability. The immune system appears to play significant roles in MSI tumors, as large numbers of infiltrating immune cells are often found in these tumors [8]. Regardless of primary organ origin, metastatic MSI cancers tend to respond to the currently popular PD1/PD-L1 immune checkpoint inhibitors, whereas MSS cancers generally do not respond well [9, 10]. Such therapies, such as pembrolizumab and nivolumab, are monoclonal antibodies against PD-1 and inhibit the PD-1/PD-L1 pathway [10]. PD-1 expressed on T cells keeps these cells in check and prevents them from attacking other cells such as PD-L1 expressing tumors, whereas blocking PD-1 with antibodies allows T cells to regain their immune defense functions to eliminate cancer cells.

Immunotherapies and targeted molecular therapies have become increasingly successful precision medicine tools in cancer treatment. This kind of therapy interrupts key molecular abnormalities in cancer by targeting specific biochemical pathways central to tumor cell growth and development. Because of their molecular specificity, targeted therapies offer significant advantages over broad spectrum chemotherapy, thus causing less harm to normal cells and fewer adverse side effects in patients. On the other hand, these therapies are highly specific and personalized, being effective for certain cancers in some patients but ineffective in others. Precision molecular diagnostics, with in-depth molecular profile differentiation of patients, is therefore a prerequisite for the success of personalized cancer management.

To better characterize and stratify cancer at the molecular level, we have embarked on an intense effort to identify molecular biomarkers of colorectal and other cancers with the goal of developing more efficient risk stratification for cancer diagnosis and treatment [11–14]. By deep proteome profiling of CRC by mass spectrometry, we observed that the protein STAT1 is upregulated in a subset of CRCs. In this study, we investigated the protein STAT1 in a large well-defined cohort as a potential molecular marker for diagnostic and prognostic differentiation of CRC.

## Materials and methods

### Clinical cases and pathological data

Tissue specimens from a total of 736 clinical cases of CRC were obtained from the Precision Pathology Biobank of Memorial Sloan Kettering Cancer Center. The study was approved by the Institutional Review Board of Memorial Sloan Kettering Cancer Center. Data was acquired retrospectively and in an anonymized manner such that consent was not required. Clinical parameters including patient age, treatment history, recurrence, and survival status were retrieved from medical records. Histologic type, tumor content ratio, and other clinicopathological parameters of all samples were re-verified by gastrointestinal subspecialty pathologists.

### Tissue microarrays

Tissue microarrays were constructed from colorectal tumors according to established protocols at Memorial Sloan Kettering Cancer Center. Tissue specimens from surgical resections dated from 1981 to 2000 were fixed with formalin and embedded in paraffin blocks. Three spatially distinct 2-mm tissue cores were drilled out from each donor paraffin block and transferred to tissue array blocks using a TMA Grand Master robot (3DHistech). The cored areas included tumor tissue as well as normal mucosal tissue.

### Immunohistochemical (IHC) staining

Tissue microarray blocks were cut into 4-μm sections. Paraffin was removed with xylene immunohistochemical staining was carried out in a Leica BOND RX slide stainer using heat-induced epitope retrieval (EDTA buffer, pH 9.0) for 30 min. STAT1-specific polyclonal antibodies were used (HPA000982, 1:750, Atlas Antibodies, Sigma).

### Immunohistochemical scoring

IHC staining intensities of STAT1 were scored by a semi-quantitative approach. The total staining intensity of tumor cells was determined and assigned values of 0, 1+, 2+, or 3+ (averaging across 3 independent tissue cores for each case), corresponding to negative, weak, medium, and strong staining, respectively. For each slide, an IHC H-score (the total weighted IHC score) was calculated by multiplying the expression intensity of individual tumor areas (score, 0–3) by their relative distribution (0–100%) to total tumor area and adding these to yield a total weighted sum. IHC H-scores therefore have a theoretical range of 0–300. Cytoplasmic STAT1 staining was scored as described. Nuclear staining was assessed as positive or negative only. All tissue samples were independently scored by two pathologists. In cases of discrepancies in IHC assessment between the two pathologists, the cases were reviewed by them together to reach a consensus score.

### TCGA and CPTAC dataset analyses

The Cancer Genome Atlas (TCGA) database was searched for correlation of gene expression between STAT1 and KRAS, BRAF, CD274, MLH1, MSH2, MSH6, or PMS2. Sequencing results and relevant clinical information of a 244-case colorectal cancer cohort [15] were downloaded from cBioPortal (https://www.cbioportal.org/). To elucidate a correlation between STAT1 protein expression and mRNA expression, we downloaded protein expression data from the Clinical Proteomic Tumor Analysis Consortium (CPTAC) data portal (https://proteomics.cancer.gov/data-portal), whose cases overlapped with TCGA CRC cohort [15, 16]. 77 cases were available with both mass spectrometry-based protein expression data and mRNA transcriptomic data.

### Statistical analysis

Categorical variables were compared using Fisher’s exact test. Survival analyses were performed using the Kaplan-Meier method and compared by a log-rank test. Multivariate analyses of prognostic factors were performed with logistic regression models by using factors that showed significant difference (*p*<0.05) in univariate analyses. Statistical analyses were performed by JMP Pro 14 software (SAS).

## Results

### STAT1 expression vs. clinicopathological features

We examined a total of 736 colorectal cancer cases in this study, including a cohort of 498 cases with early stage colorectal cancer and another cohort of 238 cases with colorectal cancer of all stages. In total, 614 patients had early stage (stages I and II) colorectal cancer of (Table 1), and 122 patients had advanced stage (stages III and IV) colorectal cancer. The cohorts are evenly distributed in terms of patient gender, with 51% (372/736) males and 49% (364/736) females. The majority (91%, 669/736) of these cases had low grade (G1 and G2) tumor differentiation, while the remaining 67 cases (9%) had poorly differentiated G3 tumors. Furthermore, 79% (581/736) of these cases had intact mismatch repair and were categorized as microsatellite stable (MSS), whereas 21% (155/736) of these cases had deficient mismatch repair and microsatellite instability (MSI).

**Table 1:** STAT1 expression and clinicopathological features of early stage colorectal cancers.

	STAT1 in cytoplasm (n = 614)			STAT1 in nucleus (n = 614)		
	Low (n = 559)	High (n = 55)	p value	Negative (n = 518)	Positive (n = 96)	p value
**Gender**			0.3979			0.1827
Male	290	25		272	43	
Female	269	30		246	53	
**Age**			0.0095			0.2176
≤65	246	14		225	35	
>65	313	41		293	61	
**Histology**			0.2985			1.0000
Mucinous	47	2		42	7	
Not mucinous	512	53		476	89	
**Tumor differentiation**			<0.0001			<0.0001
G1/2	528	35		490	73	
G3	31	20		28	23	
**Location**			<0.0001			0.0013
Left	292	12		271	33	
Right	267	43		247	63	
**AJCC stage**			0.3826			1.0000
I	208	17		190	35	
II	351	38		328	61	
**Mismatch repair**			<0.0001			<0.0001
MSS	453	20		420	53	
MSI	106	35		98	43	

**Fig 1:**
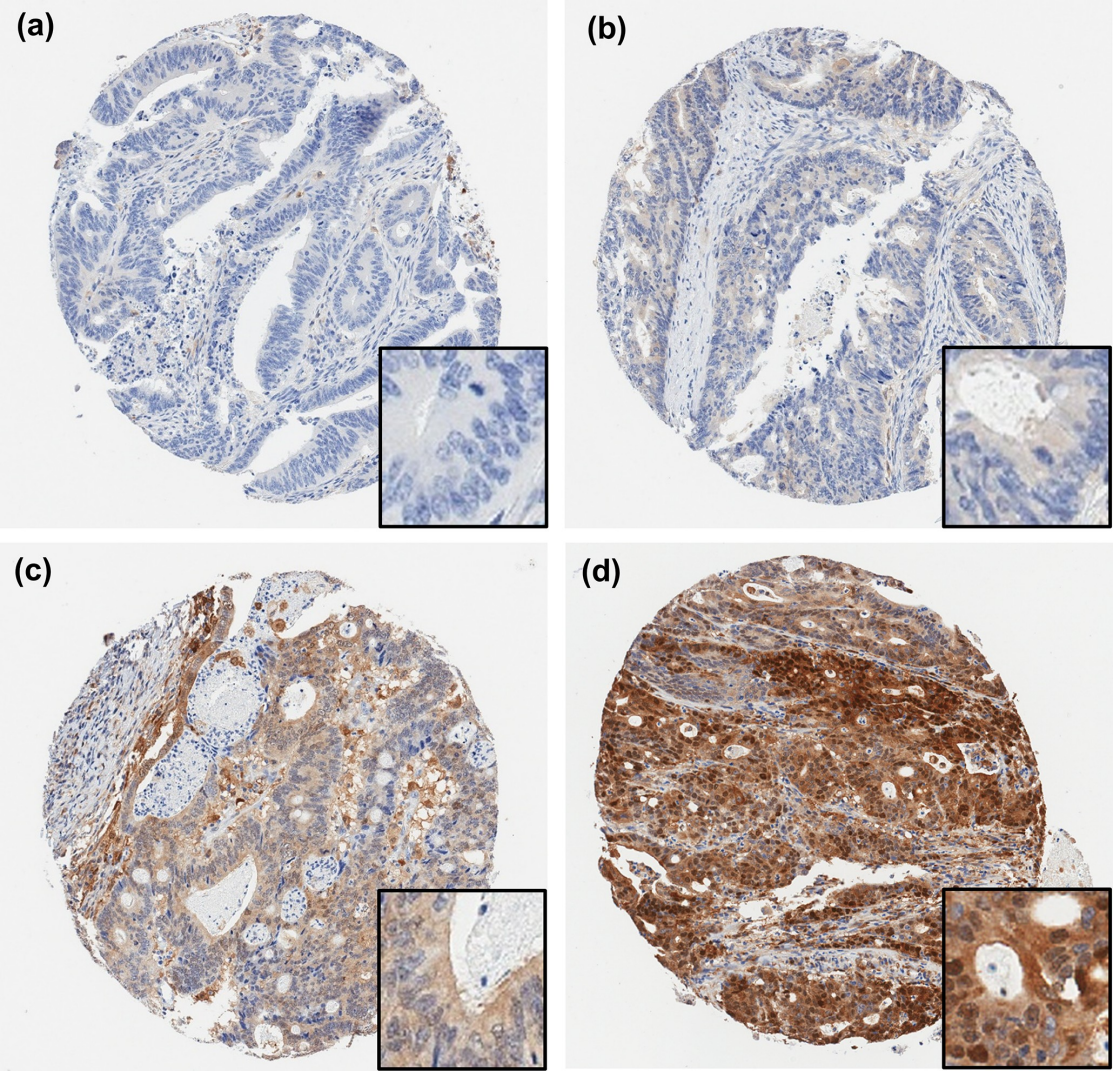
Representative tissue microarray cores showing STAT1 immunohistochemical staining. (a) Negative, (b) weakly positive, (c) moderately positive, and (d) strongly positive staining. Original magnification: 100× (insert, 400×).

The expression of STAT1 protein was examined in all 736 cases by immunohistochemistry of tissue microarrays. Representative IHC staining patterns are shown in Fig 1. STAT1 protein expression varied greatly in colon cancer samples, with levels ranging from negative or barely detectable to very high expression (Fig 1). STAT1 expression was localized mostly to the cytoplasm, but there was also expression in the nuclear compartment. Each case had a least 3 spatially distinct tissue cores evaluated, and we found that STAT1 staining intensity was very homogeneous between different cores from the same case. To study correlations between STAT1 expression and various clinicopathological features, we scored cytoplasmic STAT1 IHC staining of each case and divided the cohort into two groups, a high expression group with IHC H-scores ≥150 and a low expression group with IHC H-scores <150. Among all cases, 9% (66/736) of patients displayed high levels of cytoplasmic expression of STAT1, whereas the majority (670/736) of cases showed low levels of cytoplasmic expression. Positive nuclear expression of STAT1 was observed in 15% (101/736) of patients.

The expression level distribution of STAT1 did not differ significantly between early stage and late stage colorectal cancers, with high levels of cytoplasmic expression in 9% (55/614) of early stage cases and in 9% (11/122) of late stage cases. Nuclear staining of STAT1 was found in 11% (13/122) cases of late stage cases, which is similar to early stage cases where 16% (96/614) were positive for nuclear staining. STAT1 expression was particularly prominent in the MSI subtype. Among the 155 MSI cases from all stages, 39 (25%) cases showed high levels of cytoplasmic expression and 48 (31%) showed positive nuclear expression. Similarly, of the 141 MSI cases with early stage disease, 35 (25%) showed high cytoplasmic STAT1 protein and 43 (30%) showed nuclear STAT1 protein. These findings indicate that STAT1 expression was already elevated in early stage in a subgroup (~10%) of patients, which suggests that STAT1 could potentially serve as a marker to subtype early stage cancers.

We then performed statistical analyses to compare STAT1 expression with various clinicopathological features, including patient age and genders, tumor differentiation and location, and MSI/MSS subtype. Among the 614 cases of early stage cancer, high levels of cytoplasmic STAT1 expression were positively correlated with older age, poor tumor differentiation, right-sided location, and MSI subtype (Table 1). Nuclear expression of STAT1 was positively correlated with poor tumor differentiation, right-sided location, and the MSI subtype (Table 1). The level of cytoplasmic or nuclear STAT1 expression was not statistically different between cases that showed presence or absence of lymphovascular or perineural invasion in 498 cases for which this information was fully available (S1 Table).

### STAT1 expression vs. survival time in early stage colorectal cancer

**Fig 2:**
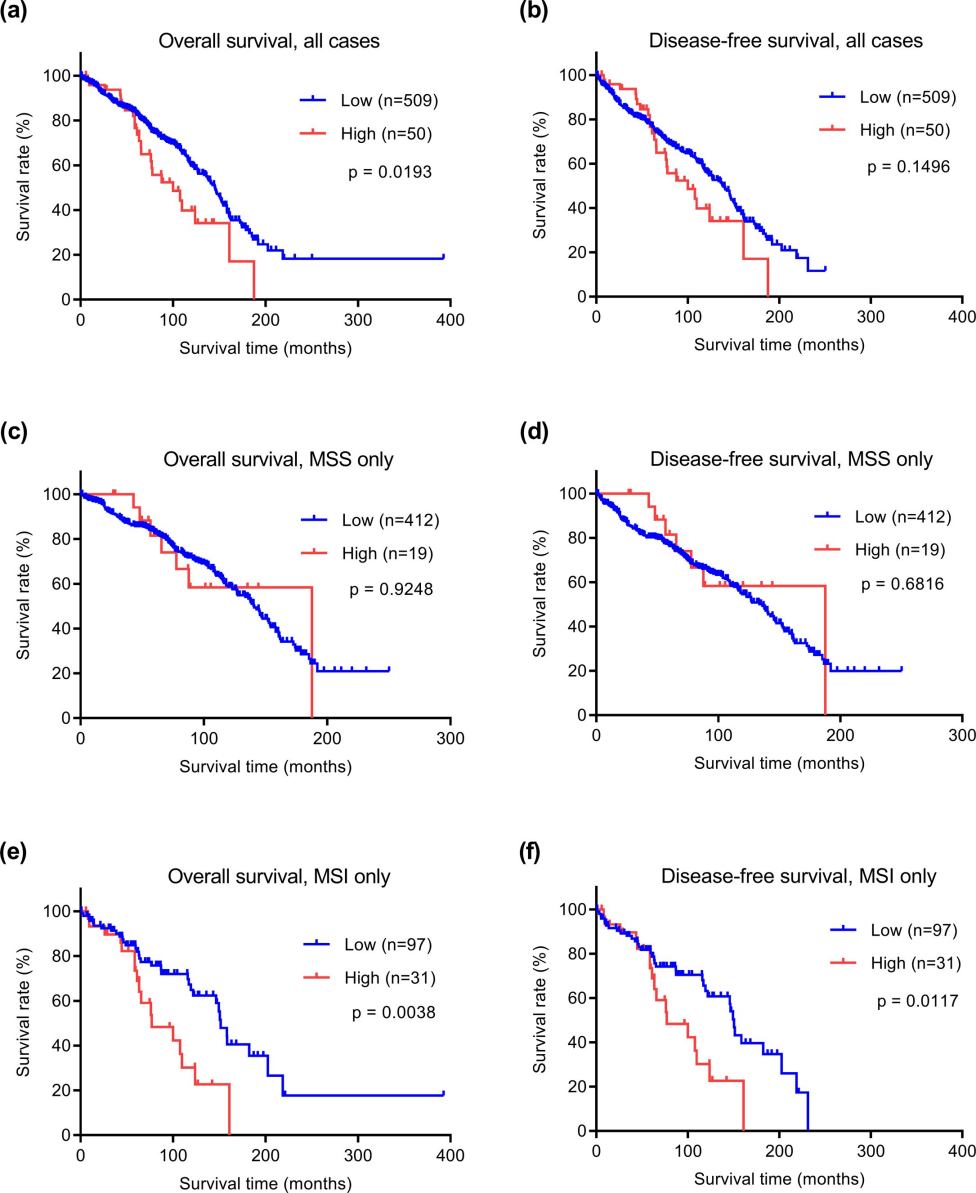
Kaplan-Meier analyses of survival of early stage colorectal cancers stratified by cytoplasmic STAT1 expression. (a, b) All 559 cases, (c, d) 431 MSS cases, and (e, f) 128 MSI cases.

**Fig 3:**
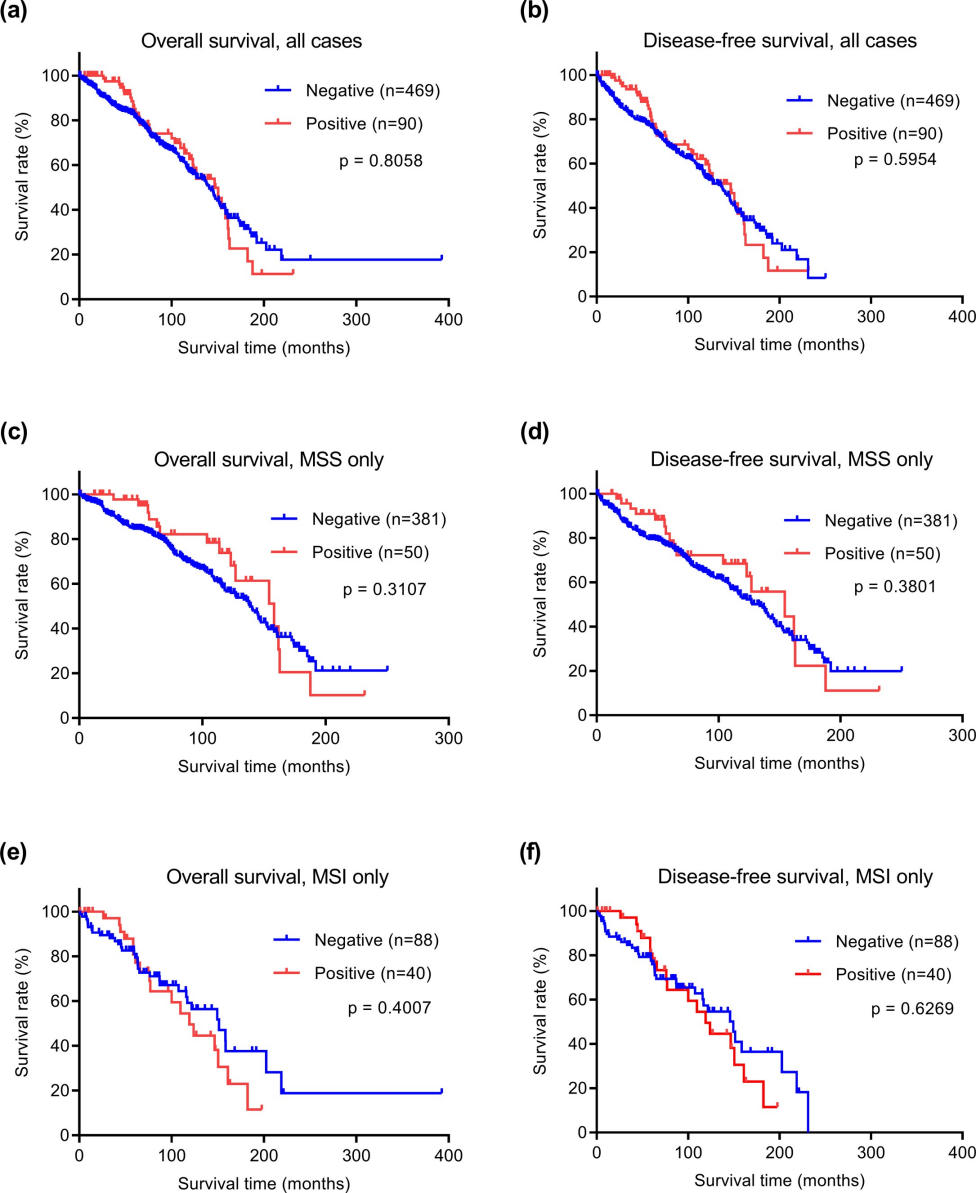
Kaplan-Meier analyses of survival of early stage colorectal cancers stratified by nuclear STAT1 expression. (a, b) All 559 cases, (c, d) 431 MSS cases, and (e, f) 128 MSI cases.

Because better subtyping of early stage colorectal cancer is particularly crucial for risk-stratifying patients prior to treatment, we examined whether STAT1 protein expression had prognostic value in early stage disease (stages I and II). Among all early stage patients, 559 cases had survival data, had not received neoadjuvant therapy, and were thus further analyzed. Mean and median clinical follow-up periods for this cohort were 80.2 and 71.9 months, respectively. Kaplan-Meier analyses were performed to examine possible correlations between STAT1 expression and clinical outcomes (disease-free and overall survival times). Patients with high cytoplasmic STAT1 expression had significantly shorter overall survival times than those with low expression (Fig 2A). The disease-free survival time also appears to be shorter for the STAT1 high expression group, although the difference is not statistically significant (Fig 2B). Next, we examined the correlation between STAT1 protein expression and survival times for both MSS and MSI subtypes separately. Among the 431 MSS cases, there was no significant difference in either overall survival or disease-free survival between STAT1 high and low groups (Fig 2C and 2D). In contrast, when the 128 MSI subtype cases were examined, we found that the STAT1 high expression group had significantly shorter overall survival and disease-free survival than the group with low expression (Fig 2E and 2F). These results reveal that high expression of cytoplasmic STAT1 correlates with shorter survival times for patients with early stage MSI colorectal cancer. In contrast to cytoplasmic STAT1, nuclear expression did not correlate with survival in early stage colorectal cancer irrespective of microsatellite status (Fig 3).

To examine whether cytoplasmic STAT1 expression is an independent prognostic factor in MSI subtype early stage colorectal cancer, we performed univariate and multivariate analyses (Table 2). Based on univariate analysis, high STAT1 expression levels correlated with shorter overall and disease-free survival times. Based on multivariate analysis, STAT1 expression was an independent indicator for overall survival. For disease-free survival, we found a similar trend, although the significance level was not reached. As would be expected, older patient age emerged as a second indicator of shorter survival in addition to STAT1 expression. Other clinical features, including tumor location, histology, tumor grade, lymphovascular, perineural, and AJCC stage, did not significantly correlate with clinical survival times (Table 2). Tumor budding was not consistently available as a scored feature for our cohort and was thus omitted from the analysis. Future work will need to test whether there exists an association between STAT1, tumor budding, and outcome. These statistical analyses further support that high expression of STAT1 is a prognostic marker for poor survival in early stage colorectal cancer of the MSI subtype.

**Table 2:** Univariate and multivariate analyses of early stage MSI colorectal cancers.

	Overall survival				Disease-free survival			
	Univariate*		Multivariate*		Univariate*		Multivariate*	
	HR (95% CI)	p value	HR (95% CI)	p value	HR (95% CI)	p value	HR (95% CI)	p value
**Gender** (male vs. female)	0.75 (0.42–1.33)	0.3319			0.77 (0.43–1.34)	0.3659		
**Age** (>65 vs. ≤65)	3.67 (1.90–7.82)	<0.0001	3.42 (1.75–7.33)	0.0002	3.37 (1.79–6.94)	<0.0001	3.37 (1.79–6.94)	<0.0001
**Tumor location** (right vs. left)	1.82 (0.86–4.48)	0.1231			1.55 (0.76–3.61)	0.2367		
**Histology** (mucinous vs. others)	0.51 (0.17–1.17)	0.1183			0.47 (0.16–1.09)	0.0835		
**Tumor grade** (G3 vs. G1/2)	1.12 (0.55–2.10)	0.7435			1.17 (0.59–2.16)	0.6305		
**AJCC stage** (II vs. I)	1.5 (0.84–2.78)	0.1704			1.43 (0.81–2.61)	0.2131		
**STAT1 expression** (high vs. low)	2.37 (1.27–4.30)	0.0075	2.03 (1.07–3.74)	0.0307	2.12 (1.14–3.80)	0.0185	1.78 (0.95–3.26)	0.0721

* Cox proportional hazards model. HR: hazard ratio. CI: confidence interval.

Using an independent TCGA dataset, we asked next whether *STAT1* gene (mRNA) expression would correlate with overall survival in early stage MSI CRC (S1 Fig) and observed a general trend in agreement with that observed in our cohort by STAT1 protein expression, especially in follow-up to ~40 months), but the small sample size of the TCGA’s MSI subgroup precludes more definitive statements. We also attempted to test whether STAT1 protein expression had any prognostic value in late stage CRC (stages III and IV). Among all late stage patients, 95 cases had survival data, had not received neoadjuvant therapy, and were thus further analyzed (S2 and S3 Figs). No statistical difference in survival was seen between either cytoplasmic or nuclear STAT1 protein expression and survival (all cases, MSS only, or MSI only, respectively). However, our statistical analysis, especially of the MSI subtype, is severely limited and under-powered by the small number of MSI cases (n = 12) in the late stage cohort.

### STAT1 vs. PD-L1, PD-1, KRAS, and BRAF status

Mismatch repair deficiency in MSI subtype tumors frequently dictates the response of these tumors to immune checkpoint inhibitors, and such immunotherapies have been approved for patients with MSI cancers. Programmed death-ligand 1 (PD-L1), encoded by CD274 gene, is an immune inhibitory ligand that is expressed on various tumor cells. Binding of PD-L1 on tumor cells to PD-1 receptors (encoded by the PDCD1 gene) on T cells blocks anti-tumor T cell activity and thus allows tumor cells to evade the host immune surveillance. Therefore, PD-L1 and PD-1 are major targets of the currently popular immune checkpoint immunotherapies. We examined whether there was a correlation between STAT1 expression and PD-L1 or PD-1 expression in colorectal cancer. We analyzed the mRNA sequencing results and clinical information of 244 colorectal adenocarcinoma cases from a TCGA colorectal cancer cohort [15].

**Fig 4:**
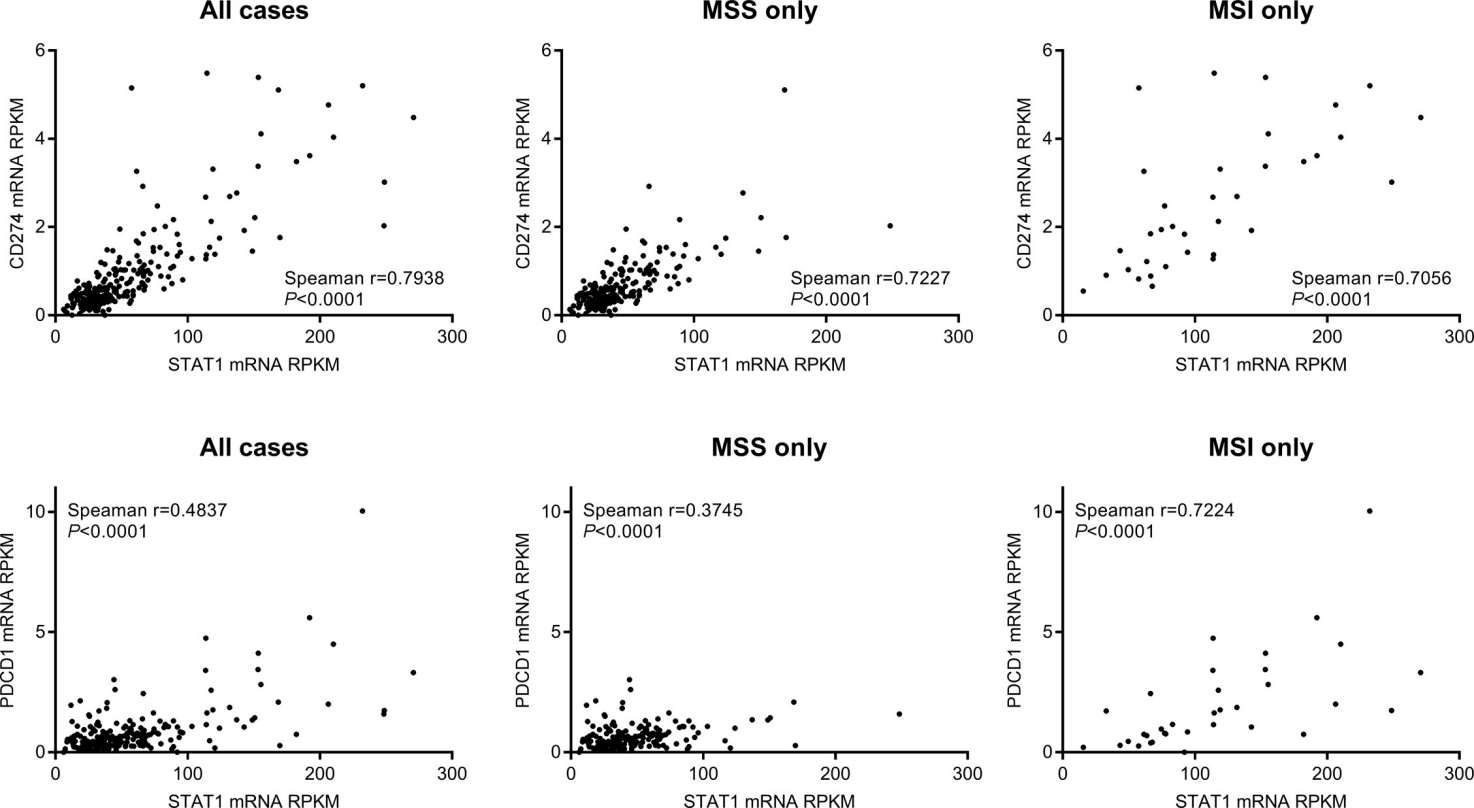
Correlations of STAT1 with CD274 (PD-L1) and PDCD1 (PD-1) gene expression using a 244-case TGCA cohort of colorectal cancer. Note the generally higher expression levels of each of the three in the MSI subtype.

This analysis revealed a significant positive correlation between the mRNA levels of STAT1 and CD274 (PD-L1) (Fig 4). The positive correlation was sustained in both MSS and MSI subtypes, although both STAT1 and CD274 expression levels appeared to be higher among MSI tumors. In contrast, STAT1 mRNA levels correlated positively but weakly with PDCD1 (PD-1) in the overall group and the MSS subgroup, yet strongly in the MSI subgroup. Again, both STAT1 and PDCD1 expression levels were higher among MSI tumors than MSS tumors.

Since KRAS and BRAF gene mutations are frequently present in colon cancers and have adverse prognostic significance, we examined whether mutations in these genes were more prevalent in tumors with high STAT1 expression, which could have contributed to poor prognosis. Using the same 244-case TCGA colorectal cancer cohort, we found that there was no significant difference in STAT1 expression levels between patients with either mutated or wild-type KRAS or mutated or wild-type BRAF (S4 and S5 Figs). This finding suggests that KRAS and BRAF mutation status were not a contributing factor to shorter survival times among patients with high STAT1 expression. Using a combined TCGA/CPTAC dataset, we observed that STAT1 mRNA expression and protein abundance correlate (S6 Fig), allowing us to approximate protein-level statements from transcriptional measurements.

### STAT1 vs. MSI diagnostic markers

Since our results suggest that STAT1 is a poor prognostic marker for MSI colorectal cancer, we investigated whether its expression correlated with any of the four standard MSI markers currently used in clinical diagnostics. MSI status in colorectal cancer is typically assessed based on the loss of expression of one or more of four mismatch repair proteins, namely, MLH1, MSH2, MSH6, and PMS2. Among 140 cases of MSI colorectal cancer in our cohort, cytoplasmic STAT1 expression correlated with MLH1 loss and intact MSH2 but not with MSH6 or PMS2 (Table 3). Nuclear STAT1 expression correlated only with MLH1 loss. In the 244-case TGCA cohort, 35 cases were of the MSI subtype. There was no correlation between STAT1 gene expression and MLH1, MSH2, MSH6, and PMS2 gene expression or mutation status of any of these four genes (data not shown). Overall there was no consistent correlation between STAT1 and the four MSI diagnostic markers, which suggests that STAT1 may have value as an additional independent marker in further sub-classification of MSI cancers.

**Table 3:** Mismatch repair marker status of early stage MSI colorectal cancers.

	Cytoplasm (n = 140)			Nucleus (n = 140)		
STAT1	Low (n = 105)	High (n = 35)	p value*	Negative (n = 97)	Positive (n = 43)	p value*
**MLH1**			0.0039			0.0336
Intact	43	5		39	9	
Loss	62	30		58	34	
**MSH2**			0.0083			0.1841
Intact	77	33		73	37	
Loss	28	2		24	6	
**MSH6**			0.3242			0.4595
Intact	57	23		53	27	
Loss	48	12		44	16	
**PMS2**			0.2400			0.3583
Intact	49	12		45	16	
Loss	56	23		52	27	

* Fisher’s exact test

## Discussion

STAT1 has been reported to play numerous roles in cancer biology. STAT1 (signal transducer and activator of transcription 1, alternatively termed transcription factor ISGF-3 components p91/p84) is a master transcription factor for IFN-related intracellular signaling. STAT molecules are phosphorylated in the cytoplasm by receptor-associated kinases, causing activation, homo- or heterodimerization, and translocation into nucleus to act as transcription factors. Specifically STAT1 can be activated by several ligands such as Interferon alpha (IFN-α), Interferon gamma (IFN-γ), Epidermal Growth Factor (EGF), Platelet Derived Growth Factor (PDGF) or Interleukin 6 (IL-6). STAT1 may act as an anti-oncogenic molecule in part by upregulation of caspases [17, 18], cyclin-dependent kinase inhibitor 1A [19], the IFN-regulatory factor 1 (IRF1)/p53 pathway [20], or downregulation of the BCL2 family [21]. On the other hand, STAT1 may act as a pro-oncogenic molecule. For example, in invasive breast carcinoma, ectopic overexpression of constitutively active STAT1 prompted the enrichment of myeloid derived suppressor cells and resulted in highly aggressive tumor growth upon transplantation into immunocompetent mice, and gene knock-down of STAT1 in tumors reversed these events and attenuated tumor progression [22]. In addition, STAT1 may exert negative impact on tumor immune surveillance. For example, STAT1 induced expression of PD-L1 upon IFN-γ stimulation in non-transformed and tumor cells [23–25], which inhibited T cell and NK cell functions [24, 25].

Given the complex functional network of STAT1 in cancer biology, it is premature for us to speculate on the precise roles of STAT1 in colorectal cancer or the MSI subtype at present. However, based on the positive correlation between STAT1 and PD-1/PD-L1 expression in MSI colorectal cancers found in this study, it is possible that STAT1 plays a pro-oncogenic role in MSI colorectal cancers. For example, STAT1 overexpression in MSI tumor cells may have induced PD-L1 overexpression, which results in an immunosuppressive microenvironment. MSI tumors in CRC typically display high levels of infiltrating CD8+ cytotoxic T lymphocytes and activated Th1 cells, which suggests that they are under great pressure of immune surveillance. However, increased expression of STAT1 may boost PD-L1/PD-1 checkpoint inhibition of T cells and allow MSI tumors to grow without immune interference, leading to worse patient survival outcomes.

Future studies will have to address mechanistic causes and roles of cytoplasmic vs. nuclear STAT1 on survival in MSI CRC. A speculative explanation for why cytoplasmic STAT1 expression may be more relevant for survival (compare Fig 2E and 2F with Fig 3E and 3F) is that the amount of cytoplasmic STAT1 accumulation may be a measure of overall STAT1 “signaling potential” (i.e., the maximum signal that may be generated upon upstream receptor-mediated activation of the cytoplasmic STAT1 protein pool). Thus cancers with high expression of cytoplasmic STAT1 may have the potential and be “primed” for very strong STAT1 signaling, whereas cancers with low cytoplasmic STAT1 would not have that capability. In addition, cytoplasmic and nuclear STAT1 may have different dynamic half-lives, perhaps causing the cytoplasmic STAT1 compartment to be the more stable to measure. Cytoplasmic and nuclear STAT1 IHC stains are not mutually exclusive (both can occur in the same cell), likely as a result of different upstream STAT signal cascade activation levels that vary between tumors. Careful analyses of these effects will require future studies.

The prognostic potential of STAT1 for cancers as reported up to date has been variable and sometimes even controversial. Correlation of STAT1 expression with better prognosis has been reported for several cancers, including colorectal cancer [26–29], esophageal cancer [30], pancreatic cancer [31], hepatocellular carcinoma [32], soft tissue sarcoma [33], and metastatic melanomas [34]. In breast cancer, correlations with good and poor prognosis have been reported for STAT1 expression [35–37], and high protein expression of STAT1 together with high levels of CD74 defined a subtype of triple negative breast cancer with increased invasiveness and metastatic potential [38]. In soft tissue sarcoma, high levels of STAT1 in tumor cells correlated with poor prognosis and metastasis [39]. We think that variable associations between STAT1 and survival reported in the literature have two principal reasons: (a) Many prior studies have analyzed survival in cohorts that comprised both early and late stage disease without taking into account that the effects of STAT1 may be stage-dependent [28]. In our current study, we also found that STAT1-based survival stratification was limited to early stage (I and II) disease, but not seen in late stage (III and IV) disease, although the late stage cohort’s analyses were limited due to small MSI subtype sample size. (b) Prior studies have been under-powered in size and composition to detect cancer subtype-specific prognostic relevance of STAT1, as is observed in our CRC cohort (MSS vs. MSI subtype).

In our study, we used a large, well-annotated clinical cohort of 614 early stage colorectal cancer patients, of which 559 patients who had not received neoadjuvant therapy were used for Kaplan-Meier survival analyses. Thus our study is characterized by an exceptionally clean clinical cohort and, due to its size, is able to test the role of STAT1 in the MSI subtype, which other studies have not been able to do. While we found that STAT1 expression as assessed by IHC for an individual patient was very homogeneous between tissue cores taken from different areas of the primary tumor, future work will need to assess whether STAT1 expression varies between, for example, the invasive edge and the center of a tumor. Our results revealed that high protein expression of STAT1 in early stage colorectal cancer, particularly of the MSI subtype, is positively correlated with shorter patient survival times, both the overall survival and disease-free survival times. Thus, STAT1 protein expression may be used as a potential risk-stratifying prognostic marker for early stage colorectal cancer, possibly guiding a decision for or against neoadjuvant therapy in stage I/II patients. Risk-stratification did not seem to extend to late stage CRC, although our late stage MSI cohort’s size was too small to definitively answer this question, and it is possible that other mechanisms (such as metastatic burden, STAT1 heterogeneity between primary and metastases, or alternative oncogenic pathways) dominate outcomes in late stage CRC.

Our study also suggests that STAT1 could be added to the routine diagnostic mismatch repair markers to further differentiate the MSI subtype of early stage colorectal cancer. Unlike MSS cancers, MSI cancers generally show positive response to the currently available immune checkpoint inhibitory therapies. However, among MSI cancers, some tumors respond well but others do not. Clearly, markers, such as STAT1, that further subgroup MSI cancers would be beneficial. Future use of digital slide scanning and computational cytoplasmic image segmentation and intensity scoring should facilitate the practical application of cytoplasmic STAT1 protein evaluation. Further extensive investigation and validation will be necessary for the routine use of STAT1 in clinical diagnostics.

## Supporting information

S1 Table Association of STAT1 expression with lymphovascular and perineural invasion in early stage colorectal cancers. (DOCX)

S1 Fig Kaplan-Meier analyses of the TCGA’s early stage CRC cohort. Overall survival stratified by *STAT1* gene (mRNA) expression (“low” expression, RPKM below median for cohort; “high” expression, RPKM at or above median for cohort).
(JPG)

S2 Fig Kaplan-Meier analyses of survival of late stage colorectal cancers stratified by cytoplasmic STAT1 expression. (a, b) All 95 cases, (c, d) 83 MSS cases, and (e, f) 12 MSI cases. Small samples size, especially of the MSI subtype, limits statistical analyses.
(JPG)

S3 Fig Kaplan-Meier analyses of survival of late stage colorectal cancers stratified by nuclear STAT1 expression. (a, b) All 95 cases, (c, d) 83 MSS cases, and (e, f) 12 MSI cases. Small samples size, especially of the MSI subtype, limits statistical analyses.
(JPG)

S4 Fig Comparison of STAT1 gene expression in MSI CRCs harboring wild-type vs mutant KRAS (three separate plots for all stages, early stage CRC, and late stage CRC, respectively). The 244-case TGCA cohort of colorectal cancer was used for this analysis.
(JPG)

S5 Fig Comparison of STAT1 gene expression in CRCs harboring wild-type vs mutant BRAF. (**a, b**) All stages, (**c, d**) early stage CRC. The 244-case TGCA cohort of colorectal cancer was used for this analysis.
(JPG)

S6 Fig Correlation between *STAT1* mRNA abundance and STAT1 protein abundance derived from a combined 77-patient TCGA/CPTAC CRC cohort, as measured by mRNA sequencing (RPKM) and protein mass spectrometry (spectrum count). (JPG)
