# Supplementary material for: STAT1 as a potential prognosis marker for poor outcomes of early stage colorectal cancer with microsatellite instability
Source: PLoS One. 2020 Apr 10;15(4):e0229252. doi: 10.1371/journal.pone.0229252 (PMC7147729; doi:10.1371/journal.pone.0229252)
Supplement: S1 Table — (DOCX) [file pone.0229252.s001.docx]

**S1 Table.** Association of STAT1 expression with lymphovascular and perineural invasion in early stage colorectal cancers

|  | **STAT1 in cytoplasm**  (n = 498) | | | **STAT1 in nucleus**  (n = 498) | | |
| --- | --- | --- | --- | --- | --- | --- |
|  | Low  (n = 459) | High  (n = 39) | p value | Negative  (n = 430) | Positive  (n = 68) | p value |
| **Lymphovascular invasion** |  |  | 0.2271 |  |  | >0.9999 |
| Absent | 424 | 34 |  | 395 | 63 |  |
| Present | 35 | 5 |  | 35 | 5 |  |
| **Perineural invasion** |  |  | >0.9999 |  |  | 0.5876 |
| Absent | 453 | 39 |  | 425 | 67 |  |
| Present | 6 | 0 |  | 5 | 1 |  |
