# Supplementary figures and images for: STAT1 as a potential prognosis marker for poor outcomes of early stage colorectal cancer with microsatellite instability
Source: PLoS One. 2020 Apr 10;15(4):e0229252. doi: 10.1371/journal.pone.0229252 (PMC7147729; doi:10.1371/journal.pone.0229252)

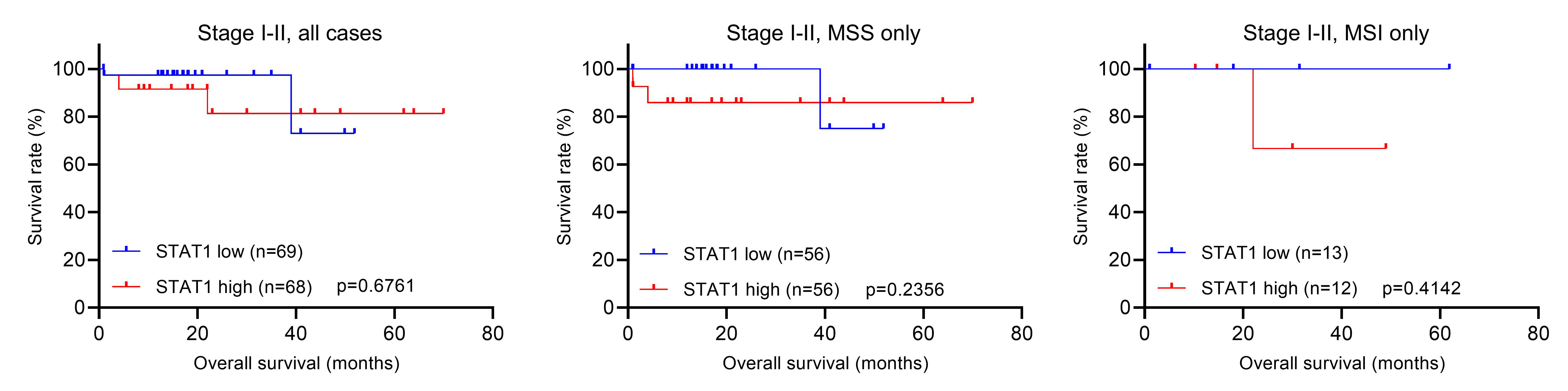

Supplement: S1 Fig — Overall survival stratified by STAT1 gene (mRNA) expression (“low” expression, RPKM below median for cohort; “high” expression, RPKM at or above median for cohort). (JPG) [file pone.0229252.s002.jpg]

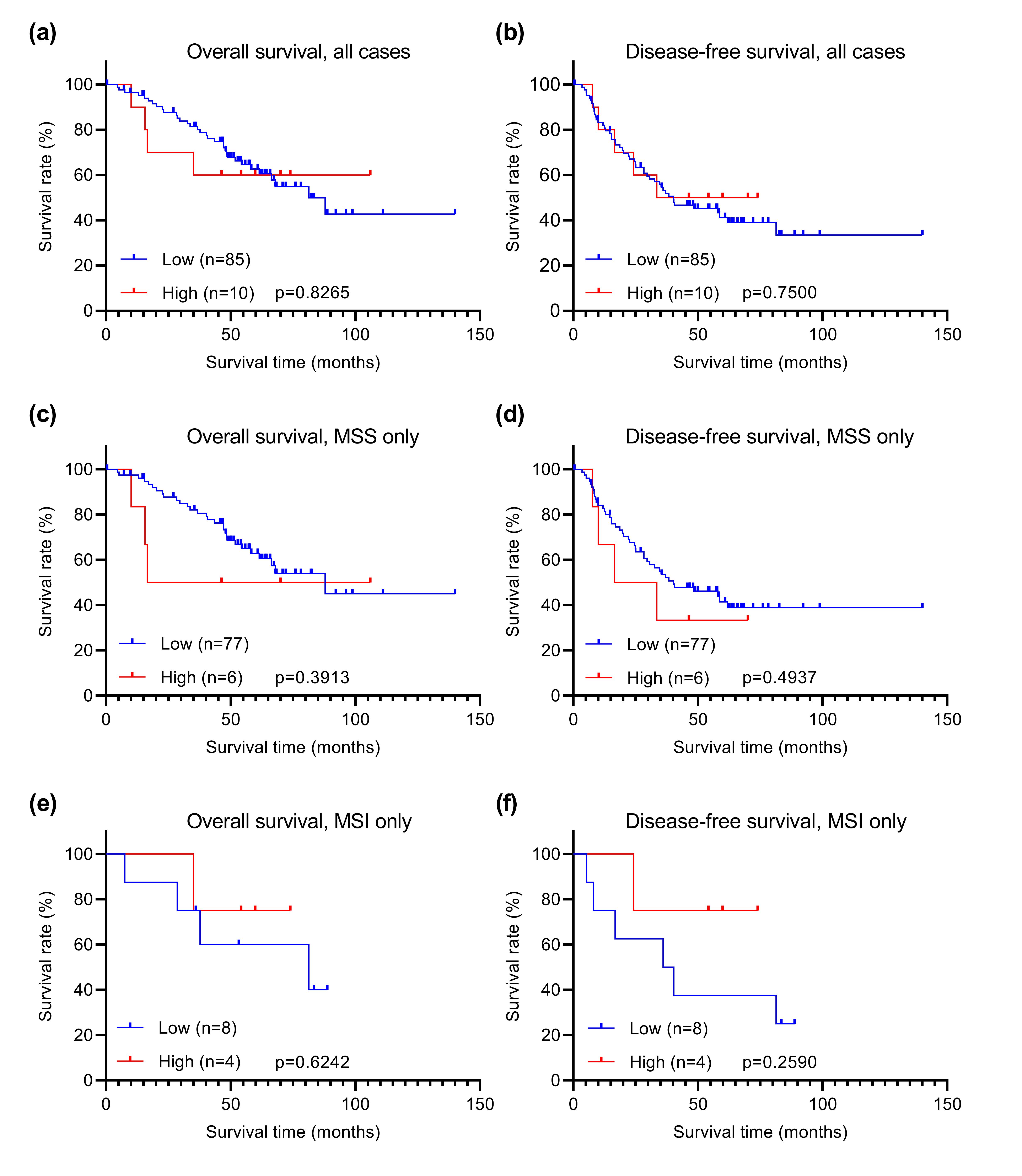

Supplement: S2 Fig — (a, b) All 95 cases, (c, d) 83 MSS cases, and (e, f) 12 MSI cases. Small samples size, especially of the MSI subtype, limits statistical analyses. (JPG) [file pone.0229252.s003.jpg]

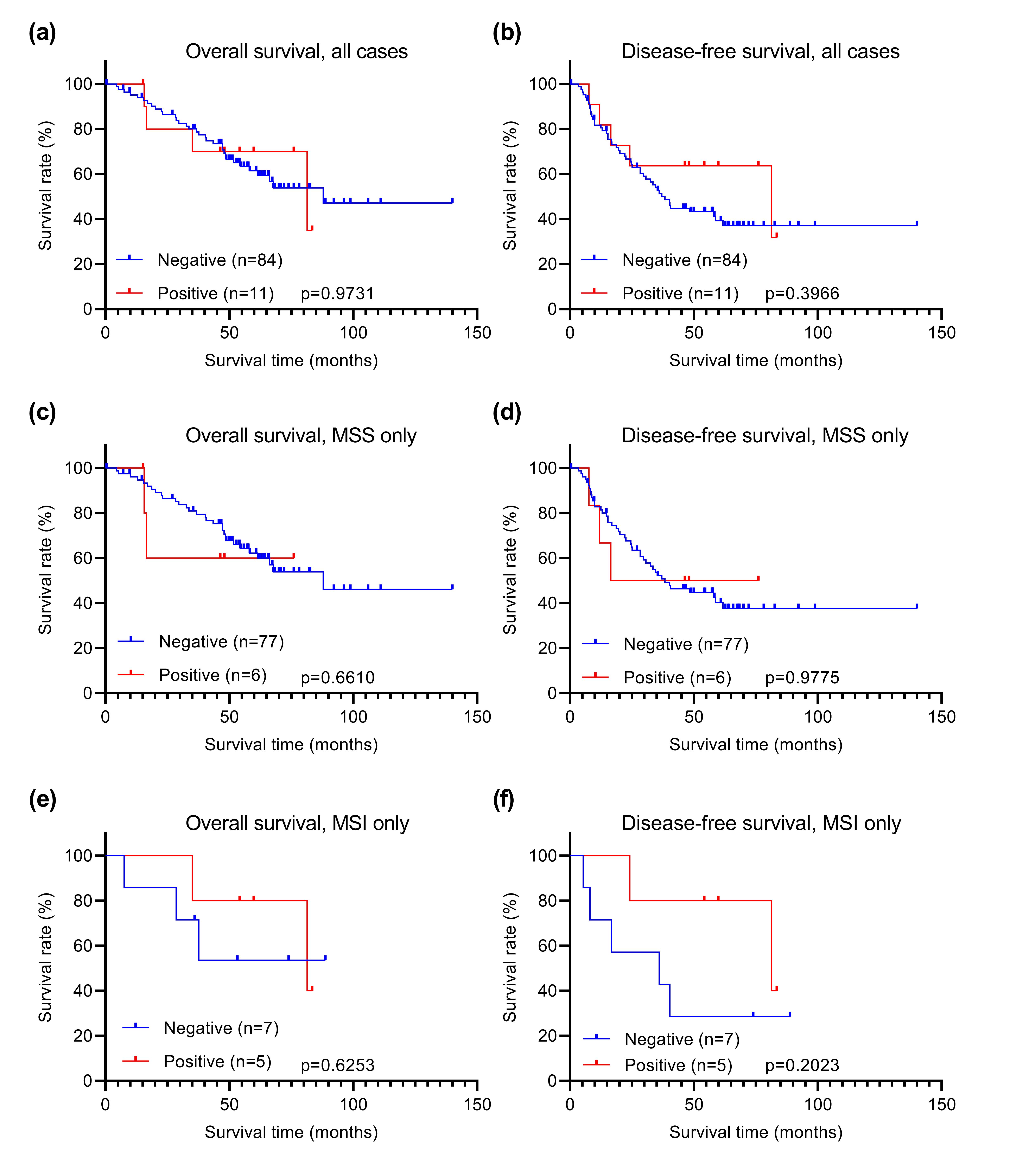

Supplement: S3 Fig — (a, b) All 95 cases, (c, d) 83 MSS cases, and (e, f) 12 MSI cases. Small samples size, especially of the MSI subtype, limits statistical analyses. (JPG) [file pone.0229252.s004.jpg]

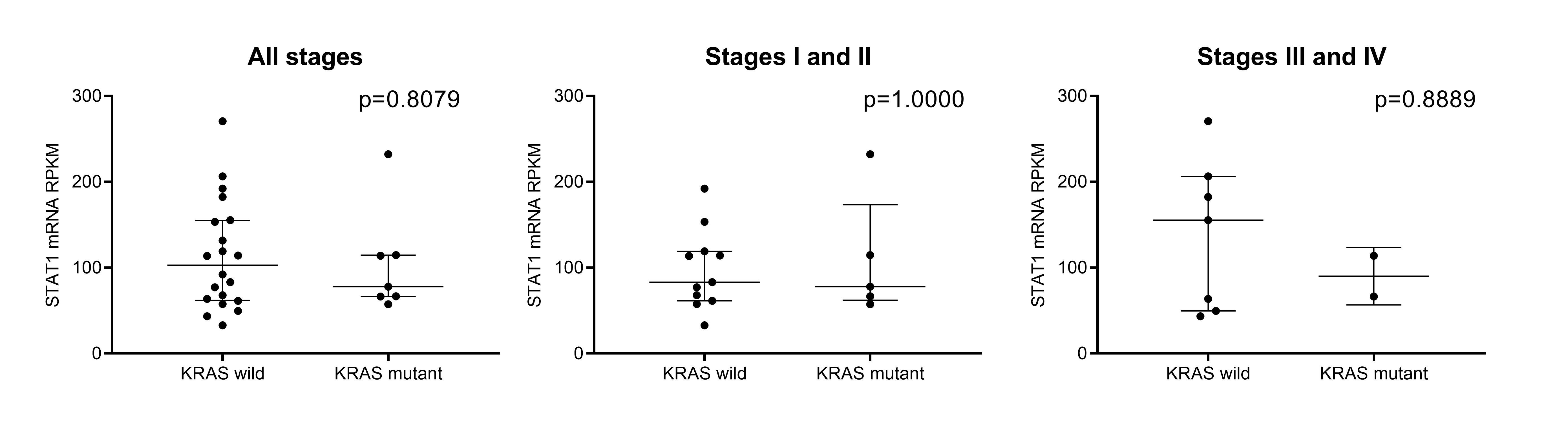

Supplement: S4 Fig — The 244-case TGCA cohort of colorectal cancer was used for this analysis. (JPG) [file pone.0229252.s005.jpg]

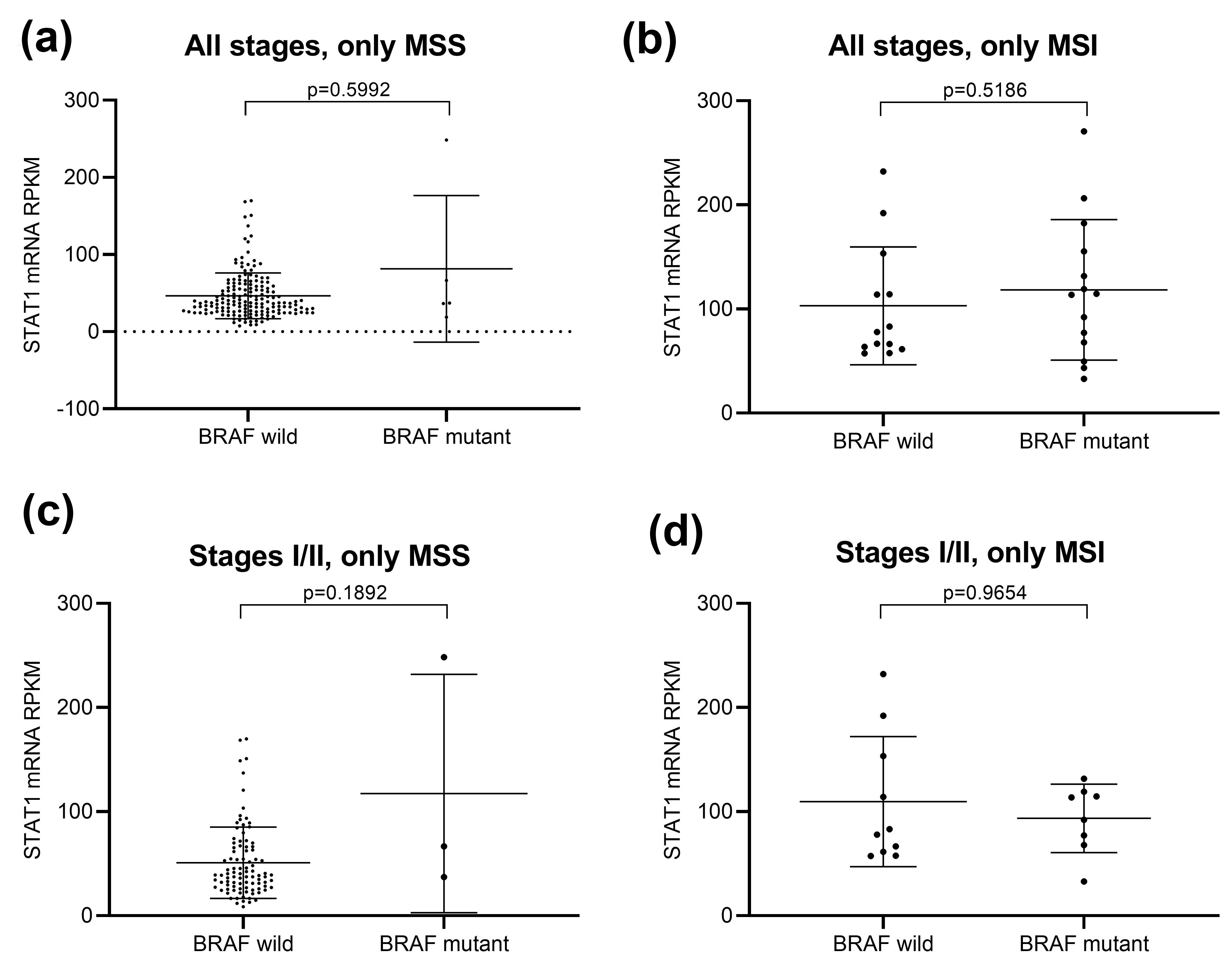

Supplement: S5 Fig — (a, b) All stages, (c, d) early stage CRC. The 244-case TGCA cohort of colorectal cancer was used for this analysis. (JPG) [file pone.0229252.s006.jpg]

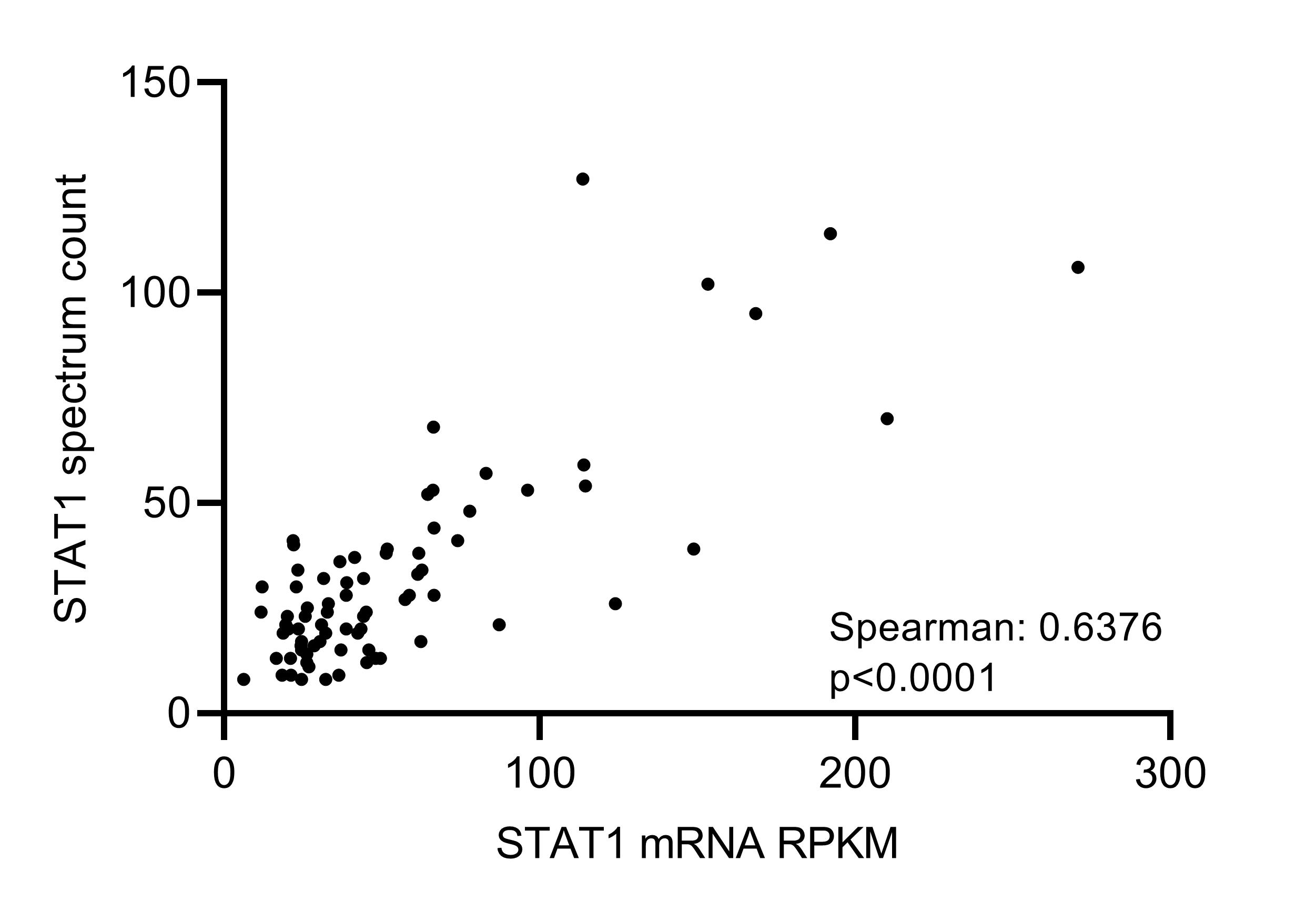

Supplement: S6 Fig — (JPG) [file pone.0229252.s007.jpg]
